# Hypertension and Exercise: A Search for Mechanisms

**DOI:** 10.5935/abc.20180146

**Published:** 2018-08

**Authors:** Bertha F. Polegato, Sergio A. R. de Paiva

**Affiliations:** Departamento de Clínica Médica - Faculdade de Medicina de Botucatu – UNESP, São Paulo, SP – Brasil

**Keywords:** Hypertension/physiopathology, Hypertension/prevention & control, Exercise, Exercise Therapy, MicroRNAs/genetics, Molecular Targeted Therapy

Arterial hypertension is a chronic disease that affects approximately 40% of the population, with higher incidence at older ages.^1^ Arterial hypertension is a risk factor for other cardiovascular diseases, such as heart failure, stroke, atherosclerosis and also chronic renal disease. It is estimated that more than 50% of deaths from coronary diseases and stroke occur in hypertensive patients;^2^ for this reason, hypertension produces high costs in health and constitutes a public health problem.^3^ In this context, the development of nonpharmacological therapies is a cost-effective strategy with few side effects, that helps in the prevention of comorbidities, such as diabetes and obesity, and increases the cardiovascular risk of the patient. Among nonpharmacological strategies, physical exercise deserves consideration.

Rodrigues et al.,^4^ in the study published in this issue of *Arquivos Brasileiros de Cardiologia*, evaluated the effect of moderate aerobic exercise on a treadmill in spontaneously hypertensive rats. The animals ran at 18-22m/min for 60 minutes, five times a week, for eight weeks.^4^ The study confirmed the anti-hypertensive effects of aerobic exercise, as already reported previously.^5^ More recently, other types of exercise in addition to aerobic training, such as resistance and interval training, have been shown to be promising in preventing hypertension.^6^ Prescription of physical exercise for the treatment and prevention of hypertension is well established, and more recent guidelines for the treatment of hypertension strongly recommend exercise as a therapeutic option.^1^^,^^2^

Even though no doubt remains about the importance of physical exercise for the management of hypertension, the mechanisms of the beneficial effects have not been fully elucidated. In this regard, the study by Rodrigues et al.^4^ proposed to investigate the transient concentration of intracellular calcium as well as the expression of microRNA (miRNA)-214, which is related to regulation of intracellular calcium and Serca-2a expression. The authors observed that physical exercise, in the presence of hypertension, increased the amplitude and decreased decay time of cytosolic calcium, which may suggest a higher availability of intracellular calcium, faster removal of this ion from the cytosol, and consequently, increased cellular relaxation. These results contribute to the understating of biological processes induced by exercises in the cardiomyocytes.

Another interesting result of the study by Rodrigues et al.^4^ was that non-hypertensive animals that underwent exercise training did not have any change in miRNA-214 expression whereas hypertensive animals that underwent training showed higher expression of this miRNA. MiRNAs are small RNA fragments that do not encode proteins, and negatively regulate gene expression at a post-transcriptional level. When discovered, miRNAs were believed to be non-functional sequences; however, since the 90’s decade, the interest in these molecules has grown and today is known to be involved in the regulation of important biological processes, including physiological and pathological ones.^7^ In hypertension, clinical and experimental studies have identified many miRNAs that may be related to the hypertension and its complications,^8^ emerging as possible biological markers and therapeutic targets in hypertension.^9^

MiRNAs constitute a complex biological control network – one miRNA can have multiple genes as targets, while one gene can be regulated by many miRNAs.^10^ So far, all possible interactions between miRNAs involved in a signaling pathway, as well as the regulatory mechanisms of miRNA functions are unknown. Maybe a miRNA expression panel is a stronger determinant than the expression of one unique miRNA in disease conditions. Despite these uncertainties, the promising role of miRNAs for the future of medicine is unquestionable, be it as a biomarker or as a therapeutic target.

Despite the results of this study, the underlying mechanisms of the beneficial effect of exercise still need to be elucidated.
